# Respectful care during childbirth in health facilities globally: a qualitative evidence synthesis

**DOI:** 10.1111/1471-0528.15015

**Published:** 2017-12-08

**Authors:** E Shakibazadeh, M Namadian, MA Bohren, JP Vogel, A Rashidian, V Nogueira Pileggi, S Madeira, S Leathersich, Ӧ Tunçalp, OT Oladapo, JP Souza, AM Gülmezoglu

**Affiliations:** ^1^ Department of Health Education and Promotion School of Public Health Tehran University of Medical Sciences Tehran Iran; ^2^ Social Determinants of Health Research Centre Zanjan University of Medical Sciences Zanjan Iran; ^3^ Department of Reproductive Health and Research, including UNDP/UNFPA/UNICEF/WHO/World Bank Special Program of Research, Development and Research Training in Human Reproduction World Health Organization Geneva Switzerland; ^4^ Department of Information, Evidence and Research Eastern Mediterranean Region World Health Organization Cairo Egypt; ^5^ Department of Health Management and Economics School of Public Health Tehran University of Medical Sciences Tehran Iran; ^6^ GLIDE Technical Cooperation and Research Ribeirão Preto São Paulo Brazil; ^7^ Department of Paediatrics Ribeirão Preto Medical School University of São Paulo Ribeirão Preto São Paulo Brazil; ^8^ Social Department of Ribeirão Preto Medical School University of São Paulo São Paulo Brazil; ^9^ King Edward Memorial Hospital for Women Subiaco WA Australia

**Keywords:** Childbirth, dignity, disrespect and abuse, health facility, hesis, qualitative evidence synt, respectful maternity care

## Abstract

**Background:**

What constitutes respectful maternity care (RMC) operationally in research and programme implementation is often variable.

**Objectives:**

To develop a conceptualisation of RMC.

**Search strategy:**

Key databases, including PubMed, CINAHL, EMBASE, Global Health Library, grey literature, and reference lists of relevant studies.

**Selection criteria:**

Primary qualitative studies focusing on care occurring during labour, childbirth, and/or immediately postpartum in health facilities, without any restrictions on locations or publication date.

**Data collection and analysis:**

A combined inductive and deductive approach was used to synthesise the data; the GRADE CERQual approach was used to assess the level of confidence in review findings.

**Main results:**

Sixty‐seven studies from 32 countries met our inclusion criteria. Twelve domains of RMC were synthesised: being free from harm and mistreatment; maintaining privacy and confidentiality; preserving women's dignity; prospective provision of information and seeking of informed consent; ensuring continuous access to family and community support; enhancing quality of physical environment and resources; providing equitable maternity care; engaging with effective communication; respecting women's choices that strengthen their capabilities to give birth; availability of competent and motivated human resources; provision of efficient and effective care; and continuity of care. Globally, women's perspectives of what constitutes RMC are quite consistent.

**Conclusions:**

This review presents an evidence‐based typology of RMC in health facilities globally, and demonstrates that the concept is broader than a reduction of disrespectful care or mistreatment of women during childbirth. Innovative approaches should be developed and tested to integrate RMC as a routine component of quality maternal and newborn care programmes.

**Tweetable abstract:**

Understanding respectful maternity care – synthesis of evidence from 67 qualitative studies.

## Tweetable abstract

Understanding respectful maternity care – synthesis of evidence from 67 qualitative studies.

## Introduction

Every day about 830 women die from pregnancy‐ or childbirth‐related complications globally. In 2015, the UN launched the Global Strategy for Women's, Children's and Adolescents’ Health, 2016–2030,^1^ with an aim to reduce the global maternal mortality ratio to fewer than 70 per 100 000 births.^2^

A central component of global efforts to reduce maternal mortality is to ensure that all women have access to skilled care before, during, and after childbirth.^3^ Access to quality services is not guaranteed for many women, however, especially in low‐ and middle‐income countries (LMICs). Even when services are available, care may be compromised by mistreatment during childbirth, including abusive, neglectful, or disrespectful care.^4^, ^5^ Several studies have identified that even if the provider is skilled in managing complications, women may refuse to seek care when they have previously experienced disrespectful care, and may also discourage others from seeking care.^4^, ^5^, ^6^, ^7^

Promoting respectful maternal care (RMC) is being increasingly recognised as a critical element of strategies to improve the utilisation and quality of maternity care,^8^ and that all women need and deserve respectful care.^9^ RMC can be defined as an approach to care that emphasises the fundamental rights of women, newborns, and families, and that promotes equitable access to evidence‐based care while recognising the unique needs and preferences of both women and newborns.^10^ The White Ribbon Alliance has defined seven domains of RMC using a rights‐based approach;^11^ however, what constitutes RMC operationally (in terms of specific behaviours, practices, or standards) in research and programme implementation is often variable. To our knowledge, no efforts have yet been made to use an evidence‐based approach to determine what constitutes RMC during childbirth in health facilities.

The aim of this qualitative evidence synthesis (QES) is to develop a conceptualisation of RMC from the perspectives of key stakeholders. The findings will support the evidence base for the related recommendations in the WHO global guideline on intrapartum care for a positive childbirth experience.

## Methods

For this QES, we followed the methodology described in the Cochrane handbook.^12^ We conducted this review in accordance with the Preferred Reporting Items for Systematic Reviews and Meta‐Analyses (PRISMA) guidelines and followed a protocol.

### Search strategy

Search strategies for PubMed, CINAHL, and EMBASE (Appendices S1, S2 and S3) were developed through the identification of all relevant terms related to childbirth, quality of care, respect, and qualitative research. Searches were conducted on 8 July 2015 and updated on 6 February 2017. We included primary qualitative studies focusing on childbirth occurring in health facilities, without any restrictions on the country's level of development, geographical locations, or publication date. We also searched the WHO Global Health Library, Cochrane Library, Database of Abstracts of Reviews of Effects (DARE), Google Scholar, Centre for Reviews and Dissemination (CRD) Database, OpenGrey, EThOS, and unpublished reports for grey literature. We contacted experts in relevant fields, and reviewed the reference lists of relevant studies to identify additional studies.

### Study selection

Two reviewers (ESh and MN) independently reviewed the titles of identified articles, and those clearly irrelevant to the topic were excluded. Abstracts of the remaining articles were reviewed for inclusion independently by two reviewers per citation (ESh, MN, JV, MB, and SL) using a screening checklist designed for this review. The full texts of all potentially eligible papers were retrieved and reviewed by two reviewers per citation (ESh, JV, MN, SL, VP, JP, and SM) based on the use of a pre‐tested eligibility checklist, including: whether the study was published in English, French, Italian, Persian, Portuguese, Spanish, or Turkish (based on the languages of the review team); whether it was a primary study; whether it used a qualitative method of data collection and analysis; whether it focused on care occurring during labour, childbirth, and/or immediately postpartum (up to 48 hours after birth); whether it primarily focused on respectful care of women; and whether it referred to births occurring at a health facility. The review included studies that evaluated the perspectives of key stakeholders within the health system, including users (women and their families), providers, administrators, and policymakers. Disagreements between reviewers during screenings were resolved by discussion with a third reviewer.

### Quality assessment

A critical appraisal form was developed using the adaptation of the Critical Appraisal Skills Program (CASP) quality assessment tool for qualitative studies (http://www.casp-uk.net). Two reviewers conducted the assessment independently (ESh, MN, VP, SM), with discussion until consensus was reached in the case of discrepancies. The findings of the critical appraisal were used for GRADE CERQual (Confidence in the Evidence from Reviews of Qualitative Research) assessments,^13^, ^14^ and interpretation of the findings.^15^

### Data extraction

Data were extracted using a standardised form developed for this review. Study characteristics, themes, authors’ interpretation, and participant quotations were extracted from the included studies.

### Data synthesis

We used a combined inductive and deductive approach to analysis. Thematic analysis methods were used to conduct initial open coding on each relevant text unit to elicit key themes emerging from the data. We also reviewed and considered existing resources to inform the organisation of a preliminary thematic framework,^16^ which included: the WHO quality of care framework for pregnant women and newborns;^17^ mistreatment of women typology;^5^ health system responsiveness domains;^18^ and the White Ribbon Alliance's^11^ seven rights of childbearing women. The preliminary coding framework was discussed iteratively, and checked against primary studies. All studies were reviewed until no new themes emerged, and agreement was reached on the definition, boundaries, and proper use of each code. During synthesis, some codes were revised and some subthemes were combined. Based on the initial coding, 12 broad themes were developed, and all text units were iteratively classified into one of the broad themes. We developed the axial coding scheme and broke up the core themes into first‐, second‐, and third‐order themes.^5^, ^19^, ^20^

To assess how much confidence can be placed in each qualitative review finding, we used the GRADE CERQual approach,^13^, ^14^ applying it to the second‐order themes as ‘high’, ‘moderate’, or ‘low’, based on the judgments made for each of the four components.

This QES is reported according to the Enhancing Transparency in Reporting the Synthesis of Qualitative Research (ENTREQ) statement.^21^

## Results

### Results of the search

**Figure 1:**
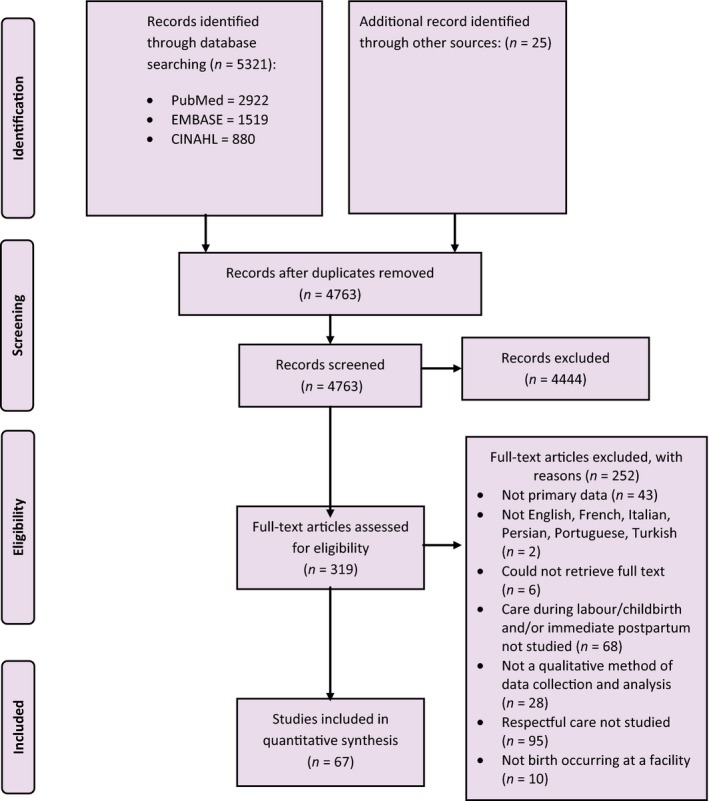
Detailed study‐selection process.

**Figure 2:**
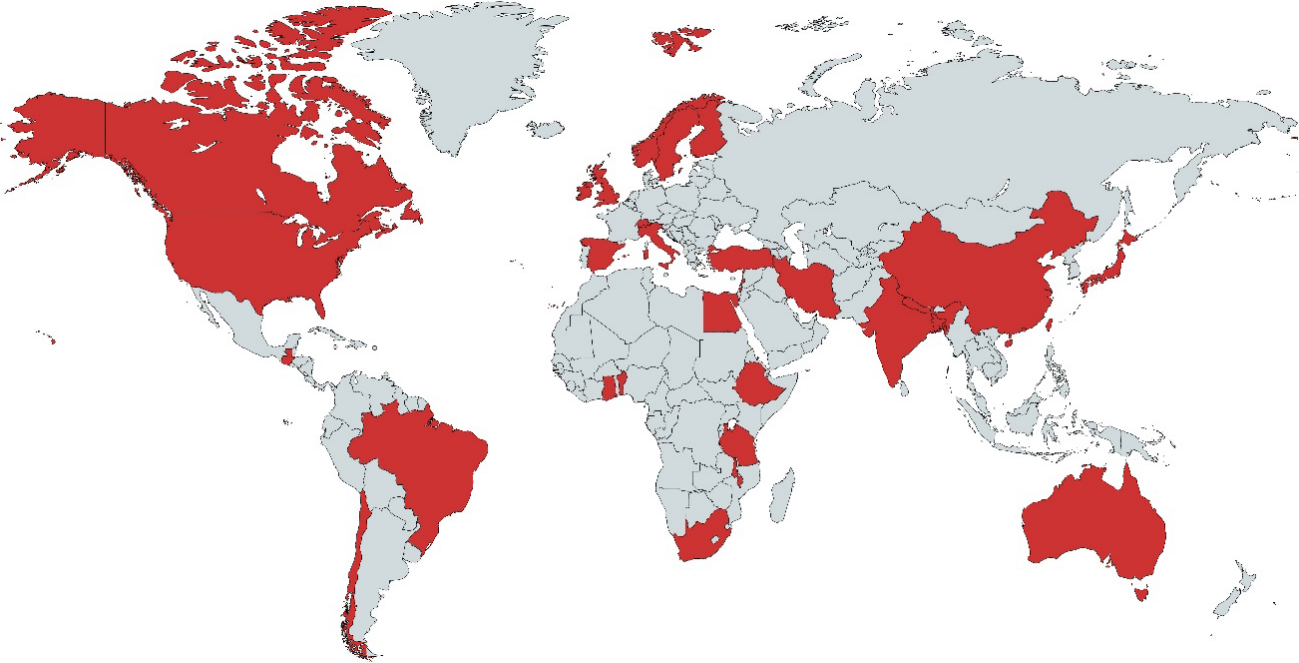
Geographical distribution of the contributing primary research countries in the analysis synthesis.

The initial and updated searches yielded 4758 citations. Full texts were retrieved for 314 potentially eligible studies. After exclusions, 67 studies were included in the review (Figure 1). This analysis synthesised findings from primary research conducted across 32 countries: six countries in sub‐Saharan Africa, seven in Asia, one in Oceania, eight in Europe, five in the Middle East and North Africa, two in North America, and three in Latin America (Figure 2). A summary of the characteristics of the included studies is presented in Table S1. Box 1 presents the 12 domains of RMC developed in the review, and Table S2 presents a typology of RMC during childbirth developed from a synthesis of the qualitative evidence. The summary of findings and the CERQual assessments are presented in Table S3. Most studies explored the experiences of women; however, many studies also included family members, midwives, obstetricians, paediatricians, nurses, facility managers, physiotherapists, midwifery students, and hospital advisory committee members as respondents.

Box 1 Twelve domains of respectful maternity care derived from the qualitative findings*

- Being free from harm and mistreatment
- Maintaining privacy and confidentiality
- Preserving women's dignity
- Prospective provision of information and seeking informed consent
- Ensuring continuous access to family and community support
- Enhancing quality of physical environment and resources
- Providing equitable maternity care
- Engaging with effective communication
- Respecting women's choices that strengthens their capabilities to give birth
- Availability of competent and motivated human resources
- Provision of efficient and effective care
- Continuity of care

*These 12 domains are the ‘third‐order themes’ from Table 2.

### Qualitative synthesis

Twelve themes emerged from the qualitative synthesis that were relevant to providing a typology of RMC during childbirth in health facilities. Many themes were homogenous across country income levels and type of participants; we have indicated where any substantive heterogeneity existed. Key findings across themes are presented below.

### Being free from harm and mistreatment

Both women and healthcare providers across countries referred to not using a loud voice when speaking to women, and having a warm and measured manner, as representing respectful care.^22^, ^23^, ^25^, ^26^, ^27^

Support from midwives enabled women to feel safe.^25^, ^26^, ^28^, ^29^, ^30^, ^31^, ^32^, ^33^, ^34^, ^35^, ^36^, ^37^, ^38^, ^39^, ^40^, ^41^ Women believed that their sense of security was facilitated by professional treatment,^42^ and by the availability of equipment and technologies.^32^ Health professionals believed that providing a safe and secure environment for women was part of humanised care.^41^

### Maintaining privacy and confidentiality

Both women and healthcare providers across the world reported maintaining privacy and confidentiality as humanised care. Women expressed a need for privacy during physical examinations and procedures,^26^, ^43^, ^45^, ^54^ by shielding them from visitors or other women,^43^, ^55^ and male staff,^24^, ^44^, ^48^, ^51^, ^52^, ^56^ and by limiting the number of staff,^24^, ^51^, ^55^ and attendants,^36^, ^48^ who are present. Healthcare providers reported that they care about women's privacy.^49^, ^50^, ^53^

Women in Malawi, Tanzania, and Nepal believed that maintaining confidentiality and ‘secrets’ about their health was a component of good‐quality care.^25^, ^26^, ^57^

### Preserving women's dignity

Women from diverse settings emphasised the importance of a positive atmosphere in the labour ward by feeling welcomed into the labour environment.^22^, ^26^, ^28^, ^35^, ^39^, ^45^, ^48^, ^55^, ^59^, ^60^

Women preferred healthcare providers that had kind attitudes, spent time with women, and were calm, tactful, warm, smiling, and caring.^28^, ^29^, ^30^, ^31^, ^39^, ^41^, ^44^, ^58^, ^63^, ^64^, ^65^ Women described their expectation to be treated as a person and not as ‘processed things’.^36^, ^60^, ^61^ To be seen as an individual – with differences and peculiarities – was expressed by women and healthcare providers as being met with respect.^31^, ^41^, ^51^, ^53^, ^56^

Respecting the cultures, values, and beliefs of women was highlighted by women and healthcare workers.^34^, ^41^, ^49^, ^51^ Women, mostly Muslims, in different countries expressed their strong preference for having a female birth attendant during labour or birth.^44^, ^48^, ^51^, ^52^

### Prospective provision of information and seeking informed consent

Women reported the need to receive information about the practice of labour, including breathing techniques, pushing, and relaxation techniques, as well as how to be prepared physically and psychologically to give birth.^26^, ^29^, ^33^, ^36^, ^37^, ^40^, ^56^, ^58^, ^61^ Healthcare providers reported that explaining the interventions that women were about to undergo was part of RMC.^41^, ^67^

Women believed that midwives should ask permission from women prior to undertaking potentially embarrassing procedures like vaginal examinations.^24^, ^44^, ^54^, ^56^ Similarly, several multi‐country studies highlighted the importance of informed consent as a component of RMC.^49^, ^53^

### Ensuring continuous access to family and community support

Most women and some healthcare providers emphasised the importance of family attendance and presence of labour companions of choice,^32^, ^33^, ^38^, ^44^, ^48^, ^50^, ^51^, ^56^, ^66^, ^70^, ^72^, ^74^ and valued it as every woman's right.^36^, ^39^, ^40^, ^64^, ^65^ Healthcare providers valued family interaction with the women and active involvement in their care.^36^, ^39^, ^40^, ^64^, ^65^, ^66^, ^67^, ^71^ In Japan, the healthcare providers and women categorised some rules and regulations as barriers to humanising birth, such as the policies restricting labour companions.^49^ The physical structure of the space was important for accommodating companions on the labour ward.^59^, ^75^

### Enhancing the quality of the physical environment and resources

Both women and healthcare providers believed that providing comfortable, clean, and calming birth environments with restricted visiting hours were conducive to promoting RMC.^22^, ^27^, ^36^, ^49^, ^51^, ^53^, ^55^, ^62^, ^64^, ^65^, ^67^, ^68^, ^73^, ^77^Healthcare providers in India and Brazil believed that to humanise birth they had to have better physical environments, including a waiting area, cleanliness, adequate bedding, and the regular supply of water and electricity, and medicines.^55^, ^56^, ^78^

Women from several countries expressed the need for adequate access to medical and non‐medical technologies, which they perceived as mechanisms to help them feel safe and reassured.^26^, ^51^, ^76^, ^79^

### Providing equitable maternity care

The availability of services for all, regardless of age, ethnicity, sexuality, religion, or other subgroups, was highlighted,^36^, ^51^, ^53^, ^80^ and treating all women equally was considered respectful.^81^ For example, several Somali‐born immigrant women in Finland were pleased with the doctors’ and nurses’ attitudes and behaviours towards them.^76^ In contrast, Somali immigrant women in Canada desired non‐judgmental care, but reported experiences of cultural discrimination.^43^

### Engaging with effective communication

Both women and healthcare providers across the world emphasised the importance of effective communication as a key component of RMC. Women appreciated receiving verbal praise and encouragement during labour, and valued the emotional support that they received from midwives.^24^, ^26^, ^34^, ^35^, ^41^, ^45^, ^51^, ^57^, ^58^, ^61^, ^66^, ^76^, ^82^, ^83^, ^86^ Healthcare providers agreed that talking and listening to the women was a critical component of humanised care,^65^, ^67^ and valued providing empathy to women.^39^, ^41^, ^53^, ^56^, ^71^, ^74^

Practicing and encouraging effective non‐verbal communication was appreciated by women and midwives.^29^, ^31^, ^48^, ^58^

Immigrant women living in developed countries highlighted the importance of the availability of interpreters because of language difficulties, and appreciated having interpreters to translate and explain.^53^, ^76^, ^84^

### Respecting women's choices that strengthen their capabilities to give birth

Respecting women's choices and empowering them was discussed across multiple settings by women,^29^, ^45^, ^55^, ^66^, ^72^ and by health professionals.^31^, ^49^, ^64^, ^65^, ^67^ Providing an opportunity for women to make decisions regarding their childbirth process was influenced by cultural contexts. Healthcare providers in Japan and women in South Africa reported that women were likely to obey the decisions made by others,^49^, ^61^ whereas in the USA, Canada, Sweden, Norway, China, Australia, Taiwan, Tanzania, and Iran women expressed strong desires to be involved in decision making.^26^, ^28^, ^29^, ^33^, ^41^, ^45^, ^46^, ^49^, ^73^, ^81^, ^87^ Midwives believed that being a good advocate was based on ensuring that women are involved in decision making,^51^, ^53^, ^70^ and considering the women's right to choose and participate in the decision‐making process.^36^, ^41^, ^65^

Encouraging free mobilisation and allowing a preferred position for birth was stated as part of humanised care by women,^45^, ^52^, ^62^, ^66^ and by healthcare providers.^64^

### Availability of competent and motivated human resources

Both the proficiency and the adequacy of staff were reported as being important in providing RMC.^28^, ^69^ Midwives’ professional knowledge and competence were considered essential by women for developing a trusting relationship.^31^, ^35^, ^43^, ^80^

The use of guidelines and protocols was discussed as potentially diminishing women's dignity in the UK by midwives, as they felt under pressure to demonstrate their compliance with guidelines.^53^ The need to gain knowledge on RMC was discussed in several studies, predominantly by healthcare providers.^49^, ^57^, ^64^, ^69^, ^75^ Supportive supervision from managers was needed to provide RMC.^65^

### Provision of efficient and effective care

Many women believed that a natural birth with minimal interventions was healthiest for themselves and for their baby,^66^ and they often wanted fewer interventions than they had received.^36^, ^43^, ^53^, ^87^ Healthcare providers in Benin believed that they should support and respect decisions made by women, and considered birth as a physiological process that does not necessarily require intervention.^67^

Women expected healthcare providers to prevent unnecessary painful interventions (e.g. minimising the use of a urinary catheter, vaginal examinations, and episiotomy). Healthcare providers believed that providing pain relief was a component of respectful care.^25^, ^26^, ^36^, ^37^, ^41^, ^45^, ^58^, ^62^, ^68^, ^72^, ^73^, ^74^, ^86^, ^88^ Women in the UK, Sweden, Italy, and Tanzania also highlighted that maternity care should be available with minimal delay.^26^, ^30^, ^37^, ^51^

### Continuity of care

Being cared for by a familiar midwife was valued by women across the world.^28^, ^36^, ^38^, ^46^, ^47^, ^49^, ^62^, ^66^, ^68^ The continuous presence of staff during and after childbirth was reassuring for most women and was requested by them.^25^, ^33^, ^34^, ^36^, ^69^, ^70^, ^73^, ^85^ Some nurses in Canada described humanised birth as ‘being with the woman and being available on demand’.^41^

Being with their babies in the facility was a stated desire for women across the globe.^40^, ^73^, ^78^, ^79^

## Discussion

### Main findings

The findings of 67 qualitative studies on the views of women, healthcare providers, and other stakeholders on what constitutes RMC were largely consistent globally. The emerging themes were used to develop a typology of RMC during childbirth in health facilities to inform further work in this important area.

Our review showed that women living in high‐income countries (HICs) tended to emphasise their rights to decision making and to active participation in their childbirth. Comparatively, women in lower‐income countries were less likely to expect personal choice and decision making over their childbirth experience. This may be attributable to differences in cultural norms around childbirth, or it could be that women in lower‐income countries were not empowered to make their own decisions. Globally, healthcare providers consistently identified the necessity of raising awareness about RMC; however, it was often described as a hard‐to‐reach target, in the face of legal and cultural pressures, particularly within cultures of blame for poor outcomes, defensive medical practices, and an over‐emphasis on documentation rather than quality of care.^53^ Healthcare providers also expressed the view that academic curricula mostly focus on biomedical care, to the exclusion of humanistic aspects of care.

### Strengths and limitations

To our knowledge, this is the first attempt to use an evidence‐based approach to develop a typology for RMC. This study used rigorous methods for synthesising and assessing the confidence of review findings.^14^ The typology can inform further work on developing evidence‐based definitions of how women experience RMC in facilities during childbirth, and how this can be measured.

These findings cannot necessarily be generalised to home birth by trained birth attendants. Moreover, new quantitative studies may add additional information related to factors affecting RMC. Two studies were excluded because of language limitation; we consider it unlikely that this has affected the overall findings.

### Interpretation (findings in light of other evidence)

Respectful maternity care (RMC) is a topic of growing attention around the world. Several recent studies have aimed to develop tools, and/or promote RMC, through applying various forms of interventions.^89^, ^90^, ^91^ A strong theoretical base is needed to inform the further development and validation of measurement tools.

This QES contributed to the framing and development of recommendations in the forthcoming WHO guideline ‘WHO recommendations on intrapartum care for a positive childbirth experience’. The domains of WHO's quality of maternal and newborn care are supported by this review.^17^, ^92^ This review further highlighted the importance of more specific themes under the domains in the WHO framework, however, including: being free from harm and mistreatment; prospective provision of information; providing equitable maternity care; and continuity of care. These themes show women's further expectations of receiving respectful care.

In Bohren's et al.^5^ systematic review on the mistreatment of women during childbirth, women reported experiences of mistreatment attributable to broader health‐system constraints or failures. Our findings also reflect this, where health‐system components (such as physical environments) mediated women's positive birth experiences. Thus improving the quality of care through promoting RMC needs to not only address interactions between the woman and the provider, but also through improvements at the health‐system level. Health‐system changes require the engagement of all health‐system actors/stakeholders, including non‐clinical staff and policymakers, to ensure that women receive the right level of care at the right time.^93^ This highlights that RMC is a broader concept than merely the absence of mistreatment, although the two are intertwined. This is important to consider when developing and evaluating interventions to promote RMC, which may not necessarily be the same as those that aim to prevent or reduce mistreatment.^8^

Interventions to promote and sustain RMC are needed at all three levels of health care (individual, health facility, and health system levels). At the individual level, several interventions are recognised as essential, rights‐based components of maternal care at birth, and need to be available to all women (such as the need for privacy and confidentiality). Others are evidence‐based interventions known to improve women's satisfaction and/or to improve the health of women or newborns, yet implementation remains limited in many settings. For example, the WHO currently recommends that all women have access to a labour companion of choice.^94^

At the health‐facility level, there is a need for measures to ensure that skilled birth attendants can provide efficient, effective, and continuous maternity care. This includes: supportive supervision, incentives, training, adequate physical infrastructure, and adequate human resources. Healthcare providers may also benefit from the more explicit inclusion of RMC themes in pre‐service and postgraduate training, although the effectiveness of training to improve RMC has not been specifically established.^95^

At the health‐system level, the creation and integration of standards and benchmarks relating to RMC should be considered. This will require the development and validation of RMC‐related indicators that along with the policy, cultural, and financial implications are adequately responsive to RMC‐related improvements.

There is evidence that improving the quality of care, including RMC, provides a return on the investment, by saving mothers and newborns.^96^, ^97^ Addressing some aspects of RMC, such as improving the physical environment, is likely to be resource intensive, and therefore the feasibility of these aspects may be limited in poorly resourced settings. Nevertheless, where RMC is a prioritised agenda within health systems, it is feasible to organise healthcare services to enable RMC across different levels.

This QES showed that the perceptions of women living in both HICs and LMICs were largely consistent, although the relative importance of the themes may vary between settings. Designing culturally appropriate interventions to promote RMC will clearly require changes in cultural norms, particularly in settings where the mistreatment of women arises from existing social norms and is regarded as acceptable. Studies show that the participatory process and sustained engagement around promoting RMC can contribute to changes in health‐facility culture.^98^

Policymakers should ensure the development and integration of written, up‐to‐date standards and benchmarks for RMC that clearly define goals, operational plans, and monitoring mechanisms. Policymakers should also be aware that shifts in health‐system infrastructure (e.g. increasing workloads) could disrupt implementation; therefore, any infrastructural changes need close monitoring to ensure the feasibility and sustainability of RMC practices.

Respectful maternity care (RMC) should not be considered as an isolated intervention but rather as a critical component for providing good‐quality care for mothers and newborns within health systems. Innovative approaches need to be developed and tested to integrate RMC into quality improvement efforts for maternal and newborn care programmes. The evaluation of RMC programmes is needed to better understand whether and how RMC can be improved in obstetric care settings, and how this can be achieved most efficiently. Such studies can provide critical components for implementation, which can then be adapted and applied in other settings. Future work should also focus on: identifying RMC indicators, in terms of validity and responsiveness in clinical settings; the effective implementation of RMC policies in different LMIC and HIC settings; and successful components/sets of components applicable in different contexts.

## Conclusion

This review presents an evidence‐based typology of the RMC during childbirth in health facilities, and demonstrates that RMC is a broader concept than merely preventing the mistreatment of women at birth. RMC can be supported and promoted at all three levels of health care (individual, health facility, and health system). Globally, women's and provider's perspectives on what constitutes RMC are fairly consistent. Further research is needed to assess the validity and responsiveness of RMC indicators before routine use in clinical settings.

### Disclosure of interests

None declared. Completed disclosure of interests form available to view online as supporting information.

### Contribution to authorship

All authors participated in the research and preparation of the manuscript, and all have reviewed and approved the manuscript as submitted and take public responsibility for it. JV, MB, AR, OTO, OT, and AMG contributed to the conception of the study; ESh, AR, JV, and MB designed the proposal; ESh wrote the first draft of the manuscript; MB, JV, MN, AR, OTO, and OT contributed to the writing of the manuscript; ESh and MN conducted the title screening; ESh, MN, JV, MB, and SL conducted the abstract screening; JV, ESh, MN, MB, SL, AR, and VP piloted the full‐text screening; ESh, JV, MN, SL, VP, JP, and SM conducted the full‐text screening; ESh, MN, VP, and SM conducted the quality assessments; ESh, MN, VP, and SM conducted the data extraction; ESh and MN assessed the confidence of the review findings; ESh, MN, MB, and JV conducted the data synthesis.

### Details of ethical approval

No ethical approval was required for this review as all data were already published in peer‐reviewed journals.

### Funding

The project was funded by the Department of Reproductive Health and Research including UNDP/UNFPA/UNICEF/WHO/World Bank Special Programme of Research, Development and Research Training in Human Reproduction, World Health Organization, Geneva, Switzerland (2015/549782‐0) and the United States Agency for International Development (USAID). The Alliance for Health Policy and Systems Research, Norwegian Agency for Development Co‐operation (NORAD), and the Research Council of Norway have provided methodological and travel support. The funders of the study had no role in the study design, data collection, analysis, interpretation, and writing of the report. The corresponding author has full access to all data in the study and has final responsibility for the decision to submit for publication.

## Supporting information


**Table S1.** Studies included in this review (authors, publication year, location, and sample characteristics).

**Table S2.** Typology of respectful maternity care during childbirth.

**Table S3.** Summary of qualitative findings and confidence assessments.

**Appendix S1.** PubMed search strategy.

**Appendix S2.** CINAHL search strategy.

**Appendix S3.** EMBASE search strategy.
