# Supplementary material for: Respectful care during childbirth in health facilities globally: a qualitative evidence synthesis
Source: BJOG. 2017 Dec 8;125(8):932–42. doi: 10.1111/1471-0528.15015 (PMC6033006; doi:10.1111/1471-0528.15015)
Supplement: Supplementary file 1 — Table S1. Studies included in this review (authors, publication year, location, and sample characteristics). [file BJO-125-932-s001.pdf]

Table S1. Studies included in this review (authors, publication year, location, and sample characteristics)

No.	Author	Year	Location	Sample characteristics
1	Proctor	1998	UK	Women and midwives
2	Behruzi et al.	2010	Japan	Women, obstetricians, pediatrician, administrative health care professor, academic midwifery professor, clinical nurse midwives, and midwifery students
3	Cheung et al.	2011	China	Women, midwives, and medical staff
4	Behruzi et al.	2014	Canada	Women, professionals, and administrators
5	O'Donnell et al.	2014	Malawi	Women and health care providers
6	Chatuluka	2015	Malawi	Women, midwives, facility managers, and hospital advisory committee members
7	Binfa et al.	2013	Chile	Women, midwives, obstetricians, and directors of maternity units
8	van Dijk et al.	2013	Guatemala	Women, midwives or comadronas, and biomedical providers
9	Rana	2014	Nepal	Women, family members, health service providers, representatives from government, professional associations/bodies, and donor organizations
10	Propst et al.	1997	USA	Nurses
11	Lundgren et al.	2002	Sweden	Midwives
12	Correa et al.	2010	Brazil	Nurses and nurses technicians
13	Souza et al.	2011	Brazil	Health professionals
14	Fujita et al.	2012	Benin	Midwives, nurse assistant, obstetricians, pediatrician, physiotherapist, other co-medical staff, and hospital director
15	Malheiros et al.	2012	Brazil	Obstetric professionals (nurses and doctors)
16	Ergin et al.	2013	Turkey	Midwives
17	Birthrights	2013	UK	Midwives and midwifery students
18	Camacho et al.	2013	Brazil	Obstetrics nurses
19	Iravani et al.	2015	Iran	Nurses
20	Bruggemann et al.	2007	Brazil	Healthcare professionals and companions
21	Woollett et al.	1990	UK	Women
22	Halldorsdottir et al.	1996	Iceland	Women
23	Kabakian-Khasholian et al.	2000	Lebanon	Women
24	Chen et al.	2001	Taiwan	Women
25	Afsana et al.	2001	Bangladesh	Women
26	Coyle et al.	2001	Australia	Women
27	Gibbins	2001	UK	Women
28	Ying Lai et al.	2002	Hong Kong	Women
29	Armellini et al.	2003	Brazil	Women
30	Hardin et al.	2004	US	Women
31	Lundgren	2004	Sweden	Women
32	Matthews et al.	2004	US	Women

33	El-Nemer et al.	2006	Egypt	Women
34	Merighi et al.	2007	Brazil	Women
35	Maputle et al.	2008	South Africa	Women
36	Gurman et al.	2008	US	Women
37	Pewitt	2008	US	Women
38	Murray et al.	2010	Australia	Women
39	Lyberg et al.	2010	Norway	Women
40	de Oliveira et al.	2011	Brazil	Women
41	Jamas et al.	2011	Brazil	Women
42	Dzomeku	2011	Ghana	Women
43	Gonçalves et al.	2011	Brazil	Women
44	Goberna-Tricas et al.	2011	Spain	Women
45	Cipolletta et al.	2012	Italy	Women
46	Kumbani et al.	2012	Malawi	Women
47	Hassan	2012	Palestine	Women
48	Maia Brasil et al.	2013	Brazil	Women
49	Nilsson	2013	Sweden	Women
50	Beake	2013	UK	Women
51	Degni et al.	2014	Finland	Women
52	Guittier et al.	2014	Switzerland	Women
53	Mensah et al.	2014	Ghana	Women
54	Karlstrom et al.	2015	Sweden	Women
55	Robertson	2015	Sweden	Women
56	Raven et al.	2015	China	Women
57	Ganle	2015	Ghana	Women
58	Clark et al.	2015	Australia	Women
59	Chalmers	2002	Canada	Women
60	Dias et al.	2006	Brazil	Women
61	Bhattacharyya et al.	2015	India	Both
62	Jha et al.	2016	India	Women
63	Ledford et al.	2016	US	Women
64	Miltenburg et al.	2016	Tanzania	Women
65	Mohale et al.	2016	Australia	Sub-Saharan African women living in Australia
66	Shifraw et al.	2016	Ethiopia	Women
67	Silva et al.	2016	Brazil	Women
