# Supplementary material for: Respectful care during childbirth in health facilities globally: a qualitative evidence synthesis
Source: BJOG. 2017 Dec 8;125(8):932–42. doi: 10.1111/1471-0528.15015 (PMC6033006; doi:10.1111/1471-0528.15015)
Supplement: Supplementary file 2 — Table S2. Typology of respectful maternity care during childbirth. [file BJO-125-932-s002.pdf]

Table S2. Typology of respectful maternity care during childbirth

Third-Order Themes	Second-Order Themes	First-Order Themes
**Being free from harm and mistreatment**	Not shouting and screaming at women	• Speaking with women in calm, measured, respectful manner
	Providing safe, supportive and secure care	• Treatment by competent professionals<br>• Providing psychosocial support to the women including praise, companionship, and empathy<br>• Providing a confident and caring relationship<br>• Collaboration between caregivers to provide a secure and continuous care<br>• A safe and secure environment
**Maintaining privacy and confidentiality**	Ensuring privacy during physical examinations and procedures	• Ensuring women are not physically exposed to others when performing vaginal or abdominal examinations<br>• Avoiding or minimizing other patients' ability to observe<br>• Shielding women from visitors of other women<br>• Where possible, accommodating women's preferences for staff gender<br>• Limiting the number of attending persons performing examinations and procedures
	Maintaining the confidentiality of women's personal information	• Maintaining confidentiality throughout all aspects of care
**Preserving women's dignity**	Giving women the feeling of being welcomed	• Providing the feeling of acceptance in the health facility<br>• Providing a positive environment that helps women to feel they are welcome in the labor environment
	Considering women as a "human being" and not a "thing"	• Providing and empowering emotional or psychological dignity or 'personhood'<br>• Seeing women as individuals with unique fears and desires, and addressing these in a respectful way<br>• Not speaking about women as an "object"
	Promoting dignified care in high- and low-risk clinical situations	• Providing personalized care for women regardless of their risk status (high- or low-risk)<br>• Transparency in providing knowledge to women with risk factors for complications<br>• Conducting vaginal examination with permission and in a gentle manner
	Respecting cultures, values and beliefs of individual women and local communities	• Providing culturally sensitive care<br>• Respecting local beliefs<br>• Providing knowledgeable healthcare that takes different women's physical and spiritual needs into account<br>• Supporting women's use of safe alternative therapies
	Being sensitive to the wishes and needs of the women	• Planning birth individually in accordance with women's wishes<br>• Being sensitive to each woman's needs
**Prospective provision of information and seeking informed consent**	Providing information about the environment, labor process/procedures and care plan clearly and neutrally	• Clear expression of what will happen during stay in health facility<br>• Providing information about birth processes and all issues relating to childbirth<br>• Providing information about how to cope with labor<br>• Providing information about breathing and relaxation techniques, ambulation, and pushing<br>• Providing information about any interventions that women are about to undergo
	Providing information about women's rights	• Providing information about rights as a patient<br>• Providing information about standard care during labor and birth
	Asking permission to carry out labor and childbirth procedures	• Asking permission to perform vaginal examinations<br>• Asking permission to physically touch the woman

	Obtaining informed consent for any interventions, and explaining the reasons for intervention or outcomes clearly	• Asking permission before performing minor procedures<br>• Providing information about available birth options and choices to enable women to make choices<br>• Obtaining informed consent for interventions like episiotomy, Cesarean section<br>• Explaining the risks and benefits of interventions or outcomes clearly
	Women informed on how to report incidences of violations or abuse	• Providing information about where to report to when incidences of violations or abuse take place<br>• Providing information about availability of counseling services for when they have experienced a violation
**Ensuring continuous access to family and community support**	Family attendance and presence of labor companions of choice	• Permitting women's companions to accompany them in labor ward (if woman desires)<br>• Welcoming the women’s partner, facility and/or friends to the facility<br>• Helping women receive support, encouragement and reassurance from labor companions<br>• Promoting companion presence by facilitating rules and regulations of the facility
	Providing space for partners, family and friends	• Providing space and comfort for partner, family and/or friends’ visits and stay
**Enhancing quality of physical environment and resources**	Providing a comfortable, clean, and calming birth environment	• Providing a comfortable and relaxing environment, supporting physical comfort and physiological needs<br>• Providing a calm, clean environment during both labor and birth<br>• Limiting ward visiting hours so women can rest
	Ensuring access to essential resources, such as medication and equipment	• Access to necessary medical investigations and medications<br>• Access to necessary medical technologies
**Providing equitable maternity care**	Availability of equitable services for all, regardless of age, ethnicity, religion, or other subgroups	• No discrimination based on ethnicity/race/religion<br>• No discrimination based on age<br>• No discrimination based on socioeconomic status<br>• No discrimination based on medical conditions<br>• No discrimination based on language<br>• Providing equal chances of receiving the same standard of dignified care for those with special needs (e.g. poor awareness of their rights, and language difficulties)
	Non-judgmental care	• Ensuring women with different ethnic, social and cultural backgrounds all received same high standard of care<br>• Providing verbal praise and encouragement during labor and birth
**Engaging with effective communication**	Talking and listening to women	• Listening to women’s concerns and trying to solve issues and addressing concerns<br>• Being sensitive to women’s wishes and needs
	Practicing and encouraging effective non-verbal communication	• Acknowledging the importance of body language, nonverbal communication, gestures, and expressions.<br>• Providing passive support by being present
	Being honest	• Valuing honesty, even in challenging situations<br>• Saying “I don’t know” and knowing when to call for help
	Availability of interpreters due to language proficiency and cultural differences	• Having interpreters to translate and explain procedures<br>• Ensuring quality of care given to women with language difficulties
	Providing empathy	• Showing compassion for what women are experiencing<br>• Presenting willingness to provide support and help by being warm, calm, patient, kind, tactful, smiling, and having a warm voice all through taking care of women<br>• Giving women full attention and true assistance<br>• Addressing women's doubts

	Facilitating and promoting women’s empowerment	• Providing trust to women<br>• Providing humanistic care with gentleness and good behaviours<br>• Ensuring that the best maternity care is done for mother and baby<br>• Empowering women by giving accurate, clear information<br>• Encouraging women to use their own emotional resources, and strengthening their self-confidence<br>• Supporting women to be in control of labor<br>• Giving women confidence in their ability as mothers
**Respecting women’s choices that strengthen their capabilities to give birth**	Encouraging active participation and decision making by women	• Facilitating women's participation in and responsibility for their childbirth<br>• Supporting and respecting women's decisions and desires
	Ensuring freedom of choice, comfort, and providing encouragement	• Implementing and upholding women’s preferences and choices<br>• Valuing and respecting women’s feelings and choices during labor and birth<br>• Defending women’s rights to control their bodies, to relieve pain, and to give birth according to her wishes
	Allowing preferred position for birth	• Allowing women to give birth in their chosen position
	Encouraging mobilization	• Allowing and encouraging women to be able to get out of bed and move around
**Availability of competent and motivated human resources**	Adequate competent staff committed to their professional responsibilities are available	• Adequate staffing levels to support women<br>• Taking responsibility for the needs of women with special needs<br>• Providing professional competence in relation to childbirth
	Capacity and awareness of professionals about humanization during childbirth	• Having a strong professional education on how to provide humanized care to women<br>• Providing continuing training for professionals about humanization during childbirth<br>• Being aware of respectful maternity care and promoting respectful care
	Supportive supervision from managers	• Making decisions to ensure provision of humanized care<br>• Playing a supervisory, monitoring and problem solving role to ensure that the facilities are operating well<br>• Taking issues related to mistreatment of women seriously, and handling reported cases with diligence<br>• Facility management resolves staff shortages<br>• Facility management takes initiative to introduce humanized care and support healthcare providers to practice humanized care<br>• Facility management positively recognize the activities of providers and staff practicing humanized care
	Promoting team-based care, involving the woman, partner, midwife, assistant nurse and other health care providers	• Building and supporting teamwork
**Provision of efficient and effective care**	Avoiding unnecessary examinations and procedures	• Not treating women as routine, or examining all women in a routine manner<br>• Not subjecting women to unnecessary procedures and interventions
	Managing pain	• Providing instructions, support and advice to allow women to find the best way/s to cope with discomfort during labor<br>• Ensuring access to pain relief methods
	Encouraging rest and recuperation after birth	• Being able to rest and recuperate from birth<br>• Postnatal ward environment that is conducive to recovery from birth and promotes breastfeeding
	Timely provision of care	• Waiting times not unnecessarily prolonged<br>• Providing on-time and continuous maternity healthcare<br>• Not refusing admission to facility or sending home without explanation
**Continuity of care**	Continuity of care throughout pregnancy and childbirth to provide focused care and advice	• Supervision of women during pregnancy and birth by a familiar midwife or same group of midwives (the named midwife)<br>• Efforts to connect women with their preferred care providers
	Continuous presence of staff throughout labor and	• Continuous presence of a health worker throughout labor and childbirth

	childbirth	• Access of women to a call button when assistance is needed<br>• Health workers available to women on demand
	Supportive care during and after the childbirth	• Promotion of mother and child bonding<br>• Respecting women's decisions in breastfeeding<br>• Providing help and support with breastfeeding in postnatal ward<br>• Providing support in caring for the baby<br>• Letting women talk to another postpartum women who has experienced childbirth

The typology presented in this table is an evidence-based classification system of how women can be respected during childbirth in health facilities, based on the findings of the evidence syntheses. The frameworks to inform development of this typology included: (1) WHO Quality of Care Framework for Pregnant Women and Newborns;[17] (2) Mistreatment of women framework;[5] (3) Health system responsiveness domains;[18] and (4) White Ribbon Alliance's 7 Rights of Childbearing Women.[11] The first-order themes are identification criteria describing specific respectful care activities. The second- and third-order themes further classify these first-order themes into meaningful groups based on common attributes. The third-order themes are ordered from the level of interpersonal relations through the level of the health system.
