# Supplementary material for: Respectful care during childbirth in health facilities globally: a qualitative evidence synthesis
Source: BJOG. 2017 Dec 8;125(8):932–42. doi: 10.1111/1471-0528.15015 (PMC6033006; doi:10.1111/1471-0528.15015)
Supplement: Supplementary file 3 — Table S3. Summary of qualitative findings and confidence assessments. [file BJO-125-932-s003.pdf]

Table S3 Summary of qualitative findings and the confidence assessments

Review Finding	Contributing studies	Confidence in the evidence	Explanation of confidence in the evidence assessment
**Being free from harm and mistreatment**			
**Not shouting and screaming at women:** Women referred to speaking with them not using a loud voice. They stated that when the health providers did not shout at them or did not speak with them harshly, they felt well received in labor ward.	(22-27)	Low	Moderate concerns about methodological limitations (4 studies with minor concerns, 1 study with moderate concerns and 1 study with serious concerns due to data collection methods). Serious concerns about adequacy of data (limited, thin data from 4 studies). Moderate concerns about relevance (studies from 5 LMIC countries: Malawi, Ghana, Tanzania, Ethiopia, and Lebanon). No concerns regarding coherence.
**Providing safe and secure care:** Women reported the importance of feeling safe and secure by receiving information, collaboration between caregivers, psychological support from the nurses including praise, companionship, and empathy, and professional treatment. Health professionals and administrators believed that providing a safe and secure environment for women was part of humanized care.	(25, 26, 28-42)	High	Moderate concerns about methodological limitations (11 studies with minor concerns, 5 studies with moderate concerns and 1 study with significant concerns due to data collection bias). Mild concerns about adequacy of data (thick data from 17 studies). Mild concerns about relevance (studies from 13 countries across multiple geographical regions and country income levels: Norway, Taiwan, Sweden, Egypt, UK, Iceland, Brazil, Italy, Australia, Malawi, Tanzania, Canada, and the US). No concerns regarding coherence.
**Maintaining privacy and confidentiality**			
**Ensuring privacy during physical examinations and procedures:** Women expressed a need for privacy. For women, privacy meant shielding them from others and limiting the number of attending persons in a facility. Midwives discussed bodily dignity by reference to protecting privacy through actions such as preventing women from being physically exposed to others, and ensuring that doors were closed. It was seen as the most obvious and relatively straightforward aspect of dignity to promote in practice.	(24, 26, 36, 43-56)	High	Moderate concerns about methodological limitations (10 studies with minor concerns, 5 studies with moderate concerns and 1 study with serious concern due to design of the study). Mild concerns about adequacy of data (thick data from 16 studies). Mild concerns about relevance (studies from 14 countries across multiple geographical regions and country income levels: Canada, UK, Hong Kong, US, Lebanon, Australia, Switzerland, Brazil, Japan, Guatemala, Palestine, India, Tanzania, and Bangladesh.) No concerns regarding coherence.
**Maintaining the confidentiality of women's personal information:** Women rated intrapartum care as good when their confidentiality was maintained. Women described confidentiality as health workers keeping secrets about birth issues.	(25, 26, 57)	Low	Mild concerns about methodological limitations (2 studies with minor concerns and 1 study with moderate concerns). Serious concerns about adequacy of data (limited, thin data from 3 studies). Serious concerns about relevance (studies from 3 LMIC: Malawi, Nepal, and Tanzania). Extent of coherence unclear due to

			limited data.
**Preserving women's dignity**			
**Giving women the feeling of being welcomed:** Women appreciated being welcomed and treated with dignity by midwives. Women placed emphasis on the importance of a positive environment that could be built partly by the women's' feeling of being welcomed into the labor environment.	(22, 26, 28, 29, 35, 39, 42, 44, 45, 48, 55, 58-60)	High	Mild concerns about methodological limitations (10 studies with minor concerns and 4 studies with moderate concerns). Mild concerns about adequacy of data (Thick data from 14 studies). Mild concerns about relevance (studies from 11 countries across multiple geographical regions and country income levels: Norway, US, Switzerland, Brazil, Malawi, Ghana, Iceland, China, India, Tanzania, and Taiwan). No concerns regarding coherence.
**Considering women as a "human being" and not a "thing":** To be seen as an individual was expressed by the healthcare providers as being met with respect and seen for oneself. They believed that having a human approach was to consider the other person, and all the differences and peculiarities which were connected with this particular person, as a human being. Women felt that being spoken to as an "object" made them anxious; they expected to be treated as a person not "processed things".	(31, 36, 41, 51, 53, 60, 61)	Moderate	Mild concerns about methodological limitations (3 studies with minor concerns and 4 studies with moderate concerns). Moderate concerns about adequacy of data (Moderately thick data from 7 studies). Moderate concerns about relevance (studies from five countries: UK, Brazil, Sweden, South Africa, and Canada). No concerns regarding coherence.
**Promoting dignified care in high- and low-risk clinical situations:** Women believed that high risk (those with special needs, with lower awareness of their rights and women who did not have English as a first language) were likely to receive poorer quality care. Midwives discussed how poor attitudes to women with high BMIs were demonstrated in how they were spoken about by staff. Health administrators believed that when it came to high-risk pregnancies, care should be even more personalized and the specific needs of a person facing such difficulties should be met. Lack of transparency in providing knowledge to a high-risk woman was considered as a barrier to humanized birth.	(41, 44, 53, 56, 62)	Moderate	Moderate concerns about methodological limitations (4 studies with minor concerns, 1 study with moderate concerns). Moderate concerns about adequacy of data (Moderate data from 5 studies). Moderate concerns about relevance (studies from 4 HICs and 1 LMIC: Canada, UK, Australia, Hong Kong, and India). Extent of coherence unclear due to limited data.
**Respecting cultures, values and beliefs of individual women and local communities:** Midwives believed that care should be culturally sensitive and tailored for and responsive to women's needs. Healthcare providers believed that their speech, approach, and care should be adapted to each person. Women, mostly Muslims, expressed their strong preference for having a female birth attendant during labor or birth, and suggested that doctors and midwives could incorporate this into their practice so as to address their concerns.	(24, 34, 41, 44, 48, 49, 51, 52, 59, 63)	High	Moderate concerns about methodological limitations (7 studies with minor concerns and 3 studies with moderate concerns). Mild concerns about adequacy of data (thick data from 10 studies). Mild concerns about relevance (studies from 8 countries across multiple geographical regions and country income levels: UK, Ghana, Canada, Bangladesh, Lebanon, Hong Kong, Switzerland, and Japan). No concerns regarding coherence.
**Being sensitive to the wishes and needs of the women:** Women appreciated individual caregivers that took them and their worries seriously**.** Obstetricians believed that having a human approach is to consider the other person and all the differences and peculiarities, which are connected with this particular person, a human being. Midwives believed that they should understand women's wishes	(28, 30, 31, 41, 64, 65)	Moderate	Moderate concerns about methodological limitations (3 studies with minor concerns and 3 studies with moderate concerns). Moderate concerns about adequacy of data (Moderate data from 6 studies). Moderate concerns about relevance (studies from 4 countries: Norway,

and try to resolve them.			Sweden, Canada, and Brazil). Moderate concerns regarding coherence.
**Prospective provision of information and seeking informed consent**			
**Providing information about the environment, labor process/procedures and care plan clearly and neutrally:** Women reported the need to receive information about birth processes and all issues relating to childbirth, and a clear expression of what is going to happen in hospital. Midwives explained to women and their family how to deal with labor. Women wanted to be taught about the practice of labor, including breathing and relaxation techniques, positioning, ambulation, and pushing, progress of labors, and physical and psychological preparedness. Healthcare providers also indicated that explaining the interventions was part of a humanistic approach to childbirth and that the humanized care was just a clear expression of what they had been doing at the hospital.	(26, 29, 33, 36, 37, 40, 41, 48, 56, 58, 61, 65-68)	High	Moderate concerns about methodological limitations (9 studies with minor concerns, 4 studies with moderate concerns and 2 studies with serious concerns due to sampling and data collection methods). Mild concerns about adequacy of data (thick data from 15 studies). Mild concerns about relevance (studies from 11 countries across multiple geographical regions and country income levels: Brazil, US, Switzerland, South Africa, Benin, Italy, Sweden, Taiwan, India, Tanzania, and Canada). No concerns regarding coherence.
**Providing information about women's rights:** Some women were not aware of their rights and continued using poor quality facility services because they regarded serious rights violations as a normal occurrence in a facility setting; however, healthcare providers believed the need to provide the best available service, provide classes on patient rights, possess and apply knowledge pertaining to medical ethics such as good service, service acceptability, availability of counseling services, and showing respect.	(57, 69)	Low	Serious concerns about methodological limitations (1 study with moderate concerns and 1 study with serious concerns due to insufficient information about methodology). Serious concerns about adequacy of data (limited, thin data from 2 studies). Serious concerns about relevance (studies from 2 LMICs: Malawi and Nepal). Moderate coherence.
**Asking permission to carry out labor and childbirth procedures:** The practice of vaginal examination and touching of the genital area was viewed by several women as unnatural, constituting an intrusion of the woman's integrity. Women believed that healthcare providers should ask permission from women for doing embarrassing procedures like pubic shaving and enemas.	(24, 44, 49, 53, 54)	Moderate	Moderate concerns about methodological limitations (3 studies with minor concerns, 1 study with moderate concerns and 1 study with serious concerns due to design of the study). Moderate concerns about adequacy of data (Moderately thick data from 5 studies). Moderate concerns about relevance (studies from 5 countries: Hong Kong, Lebanon, Japan, UK, and Palestine). No concerns regarding coherence.
**Obtaining informed consent for any interventions, and explaining the reasons for intervention or outcomes clearly:** Healthcare providers concurred that explaining to women the interventions that they are about to undergo would largely facilitate humanized birth care.	(49, 53)	Low	Moderate concerns about methodological limitations (1 study with minor concerns and 1 study with moderate concerns). Serious concerns about adequacy of data (relatively thin data from 2 studies). Serious concerns about relevance (studies from 2 HICs: UK, Japan). No concerns regarding coherence.
**Women informed on how to report incidences of violations or abuse:** Most women seeking maternity care did not know where to report to when incidences of violations or abuse took place.	(69)	Low	Serious concerns about methodological limitations (1 study with serious concerns due to insufficient information regarding methodology). Serious concerns about adequacy of data (limited, thin data from 1 study). Serious concerns about relevance (study from 1 LMIC: Malawi). Extent of coherence unclear due to limited data.
**Ensuring continuous access to family and community support**			

**Family attendance and presence of labor companions of choice:** Most women preferred presence of a labor companion; however, in some countries (e.g. Lebanon), some women were reluctant to have their partner present in the birth room due to feeling uncomfortable or shy in the presence of their partner. Women appreciated hospitals where partners were welcomed. Women felt that their partners were their main source of support and they identified support, encouragement and reassurance from them and the midwife, as being invaluable in helping them cope with their labors. Women stated they were emotionally comforted by the mere presence of their spouse and other trusted individuals. Some women indicated that they would prefer the presence of a companion, but were not informed that this was possible. Healthcare providers and women described that existing hospital rules and regulations are often a barrier to humanized care, particularly regarding restrictions on birth companions. Healthcare providers believed that the presence of a labor companion had a positive impact on the woman.	(24, 32, 33, 36-40, 44, 48-51, 55, 56, 61, 62, 64-67, 70-74)	High	Moderate concerns about methodological limitations (17 studies with minor concerns, 6 studies with moderate concerns and 3 serious concerns due to data collection methods, insufficient information on methods). Mild concerns about adequacy of data (thick data from 26 studies). Mild concerns about relevance (studies from 17 countries across multiple geographical regions and country income levels: UK, Egypt, Sweden, USA, Benin, Japan, Australia, Hong Kong, Lebanon, Brazil, Iran, Switzerland, South Africa, Guatemala, Italy, India, and China). No concerns regarding coherence.
**Providing space for partners, family and friends:** Women believed that the physical structure of the facility can act as a facilitator of humanized care. Healthcare providers reported encouraging family, especially the partner, to come to the facility and spend time with the woman. They reported providing a bed for the father or children if they want to stay at night.	(59, 75)	Low	Moderate concerns about methodological limitations (1 study with minor concerns and 1 study with moderate concerns). Serious concerns about adequacy of data (limited, thin data from 2 studies). Serious concerns about relevance (studies from 2 countries: Chile and Ghana. Serious concerns regarding coherence.
**Enhancing quality of physical environment and resources**			
**Providing a comfortable, clean, and calming birth environment:** Women valued physical comfort provided by support persons and/or by medical personnel. Providing comfortable environment/meeting environmental needs; e.g., dim light and playing music were perceived to be environments more conducive to promoting positive birth experience because women had more control over their care and surroundings; and it was perceived as humanized care (women and healthcare providers' views). The definition of quality of care given to women was related to personal requirements, such as having soap and clean clothes. Women described the importance of tranquility and cleanliness of the environment during both labor and birth**.** Immigrant women expressed preferences to share rooms with women of their own culture rather than others. Women wished to have peace, privacy, and respect in labor ward environments, and liked feeling safe during birth. For them, a sense of feeling safe and secure in hospital was more important than an attractive environment.	(22, 27, 36, 37, 43, 45, 49, 51, 53, 55, 56, 59, 62, 64-68, 73, 76-78)	High	Moderate concerns about methodological limitations (14 studies with minor concerns, 7 studies with moderate concerns and 1 study with serious concerns due to sampling methods). Mild concerns about adequacy of data (thick data from 22 studies). Mild concerns about relevance (studies from 14 countries across multiple geographical regions and country income levels: UK, US, Malawi, Finland, Brazil, Iran, Benin, Japan, Ghana, Canada, India, Ethiopia, Australia, and Italy). No concerns regarding coherence.
**Ensuring access to essential resources, such as medication and equipment:** Most of the women saw health technology in a positive light that helped them to feel safe and reassured; however, this opinion was not shared by all women. Some women did not feel that there was such a need for technology; these women asked for	(22, 26, 51, 62, 69, 76, 79)	Moderate	Moderate concerns about methodological limitations (4 studies with minor concerns, 2 studies with moderate concerns and 1 study with serious concerns due to insufficient information about methodology). Moderate concerns about

less medicalized care and saw childbirth as a physiological process in which technology was not necessary. Poor infrastructure, equipment and certain mandatory procedures like episiotomy that affect the women seeking maternity care may cause women to have negative birth experiences.			adequacy of data (Moderate data from 7 studies). Moderate concerns about relevance (studies from 6 countries: UK, Finland, Australia, Spain, Tanzania, and Malawi). Serious concerns regarding coherence.
**Providing equitable maternity care**			
**Availability of equitable services for all, regardless of ethnicity, sexuality, religion, or other subgroups:** Treating women equally, regardless of their socio-economic characteristics was seen as a form of respect from most women's point of view. Healthcare providers described how an effective emotional connection involved treating each woman as an individual, with her own unique fears and desires, whilst ensuring that all women were treated equally compassionately and kindly.	(26, 36, 51, 53, 76, 80, 81)	Moderate	Moderate concerns about methodological limitations (3 studies with minor concerns and 4 studies with moderate concerns). Moderate concerns about adequacy of data (Moderately thick data from 7 studies). Moderate concerns about relevance (studies from 6 countries: Brazil, UK, Turkey, Finland, Tanzania, and China). Moderate level of coherence.
**Non-judgmental care:** Non-judgmental care from health caregivers was echoed in the request by women undergoing FGM in a study in Canada. They referred to hurtful comments by the caregivers, verbal and non-verbal expressions of surprise when the perineum was seen by their doctors, being regarded with disgust, and having no respect shown for their cultural practice as judgmental care.	(43)	Low	Moderate concerns about methodological limitations (1 study with moderate concerns). Serious concerns about adequacy of data (limited, thin data from 1 study). Serious concerns about relevance (study from 1 HIC: Canada). Extent of coherence unclear due to limited data.
**Engaging with effective communication**			
**Talking and listening to women:** Women found motivation and comfort in receiving verbal praise and encouragement during their labor and birth. They appreciated staff talking and listening to them and valued the emotional side of the social support they received from midwives. Midwives expressed their willingness to continue humanized care in terms of talking to women. To listen to women was regarded as part of being sensitive to women's wishes and needs. Listening to women included a shift of focus in childbirth; from the midwives themselves to the woman. Humanized care from midwives point of view was in part listening to the women's and family's voices.	(24, 26, 34, 35, 40, 41, 45, 49, 51, 57, 58, 61, 65-67, 76, 82, 83)	High	Moderate concerns about methodological limitations (12 studies with minor concerns, 4 studies with moderate concerns, and 2 studies with serious concerns due to sampling and data collection methods). Mild concerns about adequacy of data (thick data from 18 studies). Mild concerns about relevance (studies from 13 countries across multiple geographical regions and country income levels: Canada, Brazil, US, Benin, UK, Iceland, Sweden, Finland, Nepal, South Africa, Japan, Tanzania, and Lebanon). No concerns regarding coherence.
**Practicing and encouraging effective non-verbal communication:** Women stressed the importance of nonverbal communication, gestures, body language and expressions to provide understanding of self-care and learning about how take better care of the child at home, and to minimize the anxiety and fear, and promote security to mother and child. They reported that an effective communication could enable them to understand the message. Midwives acknowledged the importance of body language. If verbal communication was hindered, the midwives could see the condition of the woman through the expression of the woman's body. The expression of the eyes, the face, and the whole body were important signals to the midwife. The non-verbal communication through the woman's body increased as the process of childbirth proceeded.	(29, 31, 48, 58)	Moderate	Moderate concerns about methodological limitations (1 study with minor concerns, 2 studies with moderate concerns). Moderate concerns about adequacy of data (Moderate data from 4 studies). Moderate concerns about relevance (studies from 3 countries: Brazil, Taiwan, Sweden, and Switzerland). No concerns regarding coherence.

**Being honest:** Healthcare providers valued honesty, even in the most awful situations. They believed that it was much better to say that they do not know or they are worried and they would call the doctor.	(53)	Low	Moderate concerns about methodological limitations (1 study with moderate concerns). Serious concerns about adequacy of data (limited, thin data from 1 study). Serious concerns about relevance (study from 1 HIC: UK). Extent of coherence unclear due to limited data.
**Availability of interpreters due to language proficiency and cultural differences:** Immigrant women mostly appreciated having interpreters to translate and explain everything that was said to them. Healthcare providers believed that they could not get the best care for women who did not speak any English because they can't communicate their needs.	(53, 61, 76, 84, 85)	Moderate	Moderate concerns about methodological limitations (3 studies with minor concerns, 2 studies with moderate concerns). Moderate concerns about adequacy of data (Moderate data from 5 studies). Moderate concerns about relevance (studies from 4 countries: US, UK, Finland, and South Africa). Moderate coherence.
**Providing empathy:** Women reported needing their care provider help them, such as being warm, patient, kind, and having a warm voice and smile; someone who had a heart, a certain gentleness, and knew how to behave. Midwives believed they need to love the women and to get to know them by looking at their situation and that of their families, not just the condition of pregnancy and labor. Women appreciated empathy received from healthcare providers.	(27, 29, 32, 34, 35, 37, 39, 41, 45, 53, 56, 58, 61, 67, 70, 71, 73, 74, 79, 81, 86)	High	Moderate concerns about methodological limitations (13 studies with minor concerns, 7 studies with moderate concerns, and 1 study with serious concerns due to data collection methods). Mild concerns about adequacy of data (thick data from 21 studies). Mild concerns about relevance (studies from 15 countries across multiple geographical regions and country income levels: Egypt, Spain, Canada, UK, Brazil, Benin, China, Taiwan, US, Iceland, Iran, South Africa, India, Ethiopia, and Italy). No concerns regarding coherence.
**Respecting women's choices that strengthen their capabilities to give birth**			
**Facilitate women's empowerment:** According to healthcare providers, women acknowledged the fact that they wanted to participate in decision-making**.** Midwives acknowledged women's empowerment, and believed that it was important to invite the woman to participate in and be responsible for her childbirth. Midwives stated that their job was to help women understand themselves, and empower themselves. From women's point of view, humanized birth meant respect for women's decisions and desires.	(31, 49, 64, 65, 67)	Moderate	Moderate concerns about methodological limitations (3 studies with minor concerns, 2 studies with moderate concerns). Moderate concerns about adequacy of data (Moderate data from 5 studies). Moderate concerns about relevance (studies from 4 countries: Japan, Sweden, Brazil, and Benin). Moderate coherence.
**Encouraging active participation and decision making by women:** Women expressed feelings of being in charge and being involved in decision making; midwives acknowledged a style of midwifery which was authoritative in nature. Some women expressed their gratitude to the nurses for supporting their decisions and conveying their wishes to others. Seeking women's views was demonstrated as treating women with respect. Some women reported that they were likely to obey decisions made by others; while other women had strong desires to be involved in the decision making.	(26, 28, 29, 33, 41, 45-47, 49, 53, 61, 73, 81, 82, 87)	Moderate	Moderate concerns about methodological limitations (8 studies with minor concerns and 6 studies with moderate concerns). Mild concerns about adequacy of data (thick data from 14 studies). Mild concerns about relevance (studies from 12 countries across multiple geographical regions and country income levels: Sweden, Norway, UK, Taiwan, Australia, China, Japan, US, Iran, South Africa, Tanzania, and Canada). Moderate coherence.
**Ensuring freedom of choice, comfort, and providing encouragement:** Midwives provided information to women to help	(29, 36, 40, 41, 45, 48, 51, 53, 55, 65, 70, 72, 86)	Moderate	Moderate concerns about methodological limitations (7 studies with minor concerns, 5

them make choices. They believed that being a good advocate meant respecting and working to implement and uphold women's preferences and choices. Women reported that they kept well informed about the progress of their labors and included as much as possible in decision-making. Women were appreciative of the nurses' helping them to cope with labor pain and feeling of a loss of control, and believed that the nurses had fulfilled their duties. Women believed that being able to choose the mode of delivery was as part of high-quality care.			studies with moderate concerns, and 1 study with serious concerns due to sampling bias). Mild concerns about adequacy of data (thick data from 13 studies). Moderate concerns about relevance (studies from 9 high- and upper middle income countries: Sweden, UK, Taiwan, Australia, US, Canada, India, Brazil). Moderate coherence.
**Allowing preferred position for birth:** Women reported the ability to give birth in the position chosen by them as a positive birth experience. Healthcare providers stated that they could not demand a position for the birth; to do so was regarded as ignorant and a disqualification of being an obstetric care professional. The midwives reported allowing woman to choose and be comfortable if the fetal heart beat was normal.	(45, 52, 64, 66)	Low	Moderate concerns about methodological limitations (1 study with minor concerns, 2 studies with moderate concerns, and 1 study with serious concerns due to sampling bias). Serious concerns about adequacy of data (limited, thin data from 4 studies). Moderate concerns about relevance (studies from 3 countries: Brazil, US, and Bangladesh). Moderate coherence.
**Encouraging mobilization:** Women appreciated being able to get out of bed and move around. Encouraging free mobilization was described as humanized care by healthcare providers.	(62, 64, 66)	Low	Serious concerns about methodological limitations (2 studies with moderate concerns, 1 study with serious concerns due to sampling bias). Serious concerns about adequacy of data (limited, thin data from 3 studies). Serious concerns about relevance (studies from 3 countries: US, Australia, and Brazil). No concerns regarding coherence.
**Availability of competent and motivated human resources**			
**Adequate competent staff committed to their professional responsibilities is available:** Women valued professional competence of their healthcare providers. The providers' characters, knowledge, skills, proficiency, and the staff adequacy were considered essential to have a trusting relationship. Midwives explained humanization as caring, and ensuring that the best was done for mother and baby.	(26, 28, 31, 35, 43, 44, 53, 55, 56, 67, 69, 72, 78-80, 88)	High	Moderate concerns about methodological limitations (10 studies with minor concerns, 5 studies with moderate concerns, and 1 study with serious concerns due to insufficient information on methodology). Mild concerns about adequacy of data (thick data from 16 studies). Mild concerns about relevance (studies from 14 countries across multiple geographical regions and country income levels: Norway, Sweden, Brazil, Australia, Spain, Turkey, Benin, Canada, Malawi, Iceland, Hong Kong, India, Tanzania, and the UK). No concerns regarding coherence.
**Capacity and awareness of professionals about humanization during childbirth:** Obstetricians believed that the current medical education system should be changed to include training on humanization of childbirth. Health decision-makers might be aware of respectful maternity care, but there was still a need to determine whether they believed in it. Some providers might also be biased by traditional values. It was suggested that human rights activists were generally unaware of RMC as a rights-based issue. Political leaders, media and other decision makers reportedly possess little or no knowledge of respectful maternity care.	(49, 57, 64, 69, 75, 78)	Moderate	Moderate concerns about methodological limitations (2 studies with minor concerns, 3 studies with moderate concerns and 1 study with serious concerns due to insufficient information on methodology). Moderate concerns about adequacy of data (moderate data from 6 studies). Moderate concerns about relevance (studies from 5 countries: Japan, Nepal, Chile, Malawi, and

			Brazil). Moderate coherence.
**Supportive supervision from managers:** Facility managers played a supervisory, monitoring and problem solving role to ensure that the facilities were operating well. They also indicated their seriousness in handling issues related to disrespect to clients and that each reported case was handled with diligence. Hospital management teams took the initiative to introduce humanized care and supported midwives who began to practice humanized care as a way of improving hospital management. The management team positively recognized the activities of midwives practicing humanized care**.** Healthcare providers stated that if the government did not support humanized care it would be difficult to do. The support of managers and the material provided were essential.	(49, 65, 67, 69)	Low	Moderate concerns about methodological limitations (3 studies with minor concerns, 1 study with serious concerns due to insufficient information on methodology). Moderate concerns about adequacy of data (moderate data from 4 studies). Moderate concerns about relevance (studies from 4 countries: Japan, Malawi, Benin, and Brazil). Serious concerns regarding coherence.
**Promoting team-based care, involving the woman, partner, midwife, assistant nurse and other healthcare providers:** Some women believed that giving birth requires teamwork, where the woman and her partner work together with the midwife.	(33)	Low	Moderate concerns about methodological limitations (1 study with moderate concerns). Serious concerns about adequacy of data (limited, thin data from 1 study). Serious concerns about relevance (study from 1 HIC: Sweden). Extent of coherence unclear due to limited data.
**Provision of efficient and effective care**			
**Avoiding unnecessary examinations and procedures:** Most women wanted fewer interventions in birth than they had received. Healthcare providers believed that they should support and respect the decisions made by women when they were low risk, and considered that the birth was a physiological process not needing intervention and they should let the process flow naturally.	(36, 43, 44, 53, 64-67, 87)	High	Moderate concerns about methodological limitations (5 studies with minor concerns, 3 studies with moderate concerns and 1 study with serious concerns due to sampling bias). Mild concerns about adequacy of data (thick data from 9 studies). Mild concerns about relevance (studies from 6 countries: Canada, Brazil, US, Hong Kong, UK, and Benin). No concerns regarding coherence.
**Managing Pain:** Women expected that healthcare providers respected the fact that they were in pain and try to mitigate pain during labor and after birth; and prevent unnecessary painful interventions (for example: frequent use of urinary catheter and vaginal examination; routine use of episiotomy and enemas). They expected to have choices of selecting non pharmacological measures for pain relief. Healthcare providers believed that understanding women's pain and relieving it was a respectful manner.	(25, 26, 36, 37, 41, 45, 58, 62, 68, 72-74, 86, 88)	Moderate	Moderate concerns about methodological limitations (9 studies with minor concerns, 5 studies with moderate concerns). Moderate concerns about adequacy of data (moderately thick data from 14 studies). Mild concerns about relevance (studies from countries across multiple geographical regions and country income levels: Canada, Sweden, Brazil, Malawi, Tanzania, Australia, Sweden, Iran, China, US, and Italy). No concerns regarding coherence.
**Encouraging rest and recuperate after birth:** Those who had complex deliveries were more likely to complain about the limited or absent help to recover from birth and breastfeed, and the limited opportunities to rest.	(53, 85)	Low	Moderate concerns about methodological limitations (2 studies with moderate concerns). Serious concerns about adequacy of data (limited, thin data from 2 studies). Serious concerns about relevance (studies from 1 HIC: UK). No concerns regarding coherence.

**Timely provision of care:** Women did not want to wait for a long time to receive care. They stressed the importance of maternity healthcare that was available and easily accessible.	(26, 30, 36, 37, 51, 72)	Moderate	Moderate concerns about methodological limitations (3 studies with minor concerns and 3 studies with moderate concerns). Moderate concerns about adequacy of data (moderately thick data from 6 studies). Low concerns about relevance (studies from 4 high- and upper middle income countries: Brazil, UK, Sweden, and Italy and 1from a low-income country: Tanzania). No concerns regarding coherence.
**Continuity of care**			
**Continuity of care throughout pregnancy and childbirth to provide focused care and advice:** Both midwives and women placed emphasis on the need of women for attention from one particular midwife and having an ongoing relationship with their midwife, so that they received focused care and advice. Women also reported that they trusted the familiar carer above others and women perceived many positive benefits when they had the opportunity to be cared for throughout pregnancy and birth by a familiar midwife. Women appreciated the option to choose their midwives.	(28, 34, 36, 38, 41, 46, 47, 49, 51, 62, 66, 88)	High	Moderate concerns about methodological limitations (8 studies with minor concerns, 2 studies with moderate concerns and 1 study with serious concerns due to sampling bias). Mild concerns about adequacy of data (thick data from 11 studies). Mild concerns about relevance (studies from 7 high- and upper middle income countries: Brazil, UK, Canada, Norway, US, Japan, and Australia). No concerns regarding coherence.
**Continuous presence of staff throughout labor and childbirth:** Continuous presence of staff was requested by women. Women desired midwives to be with them and provide relevant care during labor and birth. Presence of midwives in the room was reassuring for most women; confirmation and support from the midwife strengthened the women's own ability to stay in control and, at the same time, enabled them to relax. Women also did not want to wait for receiving care.	(25, 32-34, 36, 37, 41, 46, 47, 49, 50, 69, 71-73)	High	Moderate concerns about methodological limitations (8 studies with minor concerns, 4studies with moderate concerns, and 2 studies with serious concerns due to insufficient information on methodology). Mild concerns about adequacy of data (thick data from 14 studies). Mild concerns about relevance (studies from 12 countries across multiple geographical regions and country income levels: Malawi, Egypt, Sweden, Australia, Japan, US, Iran, Brazil, Canada, Guatemala, Italy, and UK). No concerns regarding coherence.
**Supportive care during and after the childbirth:** Women liked to have the opportunity to talk to midwives after labor to clarify some issues and gain confidence that all was normal. Women described experiences of easy access, availability, and continuity of care with midwives. Some women experienced a lot of support in parent groups during and after childbirth. Most women stated it was a right to be able to see and hold their baby immediately after birth, and appreciated efforts to promote bonding between mother and infant. Healthcare providers believed that some women don't want to breastfeed the child and some professionals demand them to do so but acknowledge that they have to respect women's decisions. Respectful care for the newborn included minimal time away from the mother.	(25, 30, 38, 40, 41, 49-51, 70, 73, 78, 79, 83, 85)	High	Moderate concerns about methodological limitations (12 studies with minor concerns, 3 studies with moderate concerns and 1 study with serious concerns due to sampling bias). Mild concerns about adequacy of data (thick data from 16 studies). Mild concerns about relevance (studies from 7 countries across multiple geographical regions and country income levels: UK, Sweden, Australia, Brazil, Canada, Malawi, Japan, Spain, Iran, and Guatemala). No concerns regarding coherence.

A summary of the review findings from the qualitative synthesis are presented here, with the relevant studies contributing to each review finding. The confidence in the evidence refers to the overall CERQual assessment of the methodological limitations of included studies, relevance, adequacy, and

coherence, and is rated as high, moderate, or low. The explanation of the assessment of the confidence in the evidence provides a brief assessment of each CERQual domain to support the overall CERQual assessment.
