# Supplementary material for: Respectful care during childbirth in health facilities globally: a qualitative evidence synthesis
Source: BJOG. 2017 Dec 8;125(8):932–42. doi: 10.1111/1471-0528.15015 (PMC6033006; doi:10.1111/1471-0528.15015)
Supplement: Supplementary file 4 — Appendix S1. PubMed search strategy. [file BJO-125-932-s004.pdf]

Appendix S1. PubMed search strategy

Search terms shall specify the maternal/perinatal health, facility, delivery, respectful care, and study design (qualitative study).

	#	**Searches (PubMed)**
**MATERNAL / PERINATAL HEALTH/CHILDBIRTH**	1	“perinatal care”[tiab] OR “peri natal care”[tiab] OR “perinatal healthcare”[tiab] OR “peri natal healthcare”[tiab] OR “perinatal health care”[tiab] OR “peri natal health care”[tiab] OR "perinatal care"[Mesh] OR “maternal health services"[mesh] OR (“perinatal service”[tiab] OR “peri natal service”[tiab] OR “perinatal services”[tiab] OR “peri natal services”[tiab] OR “perinatal health service”[tiab] OR “peri natal health service”[tiab] OR “perinatal health services”[tiab] OR “peri natal health services”[tiab] OR “maternal care”[tiab] OR “maternal health care”[tiab] OR “maternity care” OR “maternal healthcare”[tiab] OR “maternal service”[tiab] OR “maternal health service”[tiab] OR “maternal services”[tiab] OR “maternal health services”[tiab] OR birth[tiab] OR births[tiab] OR childbirth[tiab] OR childbirth[tw] OR childbirths[tiab] OR delivery[tiab] OR deliveries[tiab]) OR “birthing centers"[tiab] OR "maternal-child health centers"[tiab] OR "delivery rooms"[tiab] OR "maternity hospitals"[tiab] OR “facility based delivery”[tw] OR “facility based deliveries”[tw] OR “facility delivery”[tw] OR “facility deliveries”[tw] OR “facility based births”[tw] OR “facility based birth”[tw] OR “facility-based childbirth”[tw] OR “facility-based child birth”[tw] OR “facility birth”[tw] OR “facility births”[tw] OR “clinic delivery”[tw] OR “clinic deliveries”[tw] OR “clinic births”[tw] OR “clinic birth”[tw] OR “hospital delivery”[tw] OR “hospital deliveries”[tw] OR “hospital birth”[tw] OR “hospital births”[tw] OR “hospital childbirth”[tw] OR “hospital childbirths”[tw] OR “hospital based deliveries”[tw] OR “hospital based delivery”[tw] OR “hospital based births”[tw] OR “institutional birth”[tw] OR “institutional births”[tw] OR “institutional childbirth”[tw] OR “institutional childbirths”[tw] OR “institutional delivery”[tw] OR “institutional deliveries”[tw]
	2	"Patient Satisfaction"[Mesh] OR "Quality of Health Care"[Mesh:NoExp] OR "Human Rights"[Mesh]
**RESPECTFUL CARE**	3	Respectful[tw] OR dignity[tw] OR dignified[tw] OR undignified[tw] OR humanis*[tw] OR medicalize[tw] OR medicalise[tw] OR "human rights"[tw] OR empower*[tw] OR "quality of care"[tw] OR companion*[tw]
**MH_ RMC**	4	#1 AND (#2 OR #3)
**Study Design**	5	“Qualitative Research"[Mesh] OR "Interviews as Topic"[Mesh] OR "Focus Groups"[Mesh] OR qualitative[tw] OR focus group*[tw] OR semi-structured[tw] OR in-depth[tw] OR “thematic analysis”[tw] or “content analysis”[tw] OR “grounded theory”[tw] OR “constant comparison”[tw]
**Search results**	6	#4 AND #5
