# Supplementary material for: Respectful care during childbirth in health facilities globally: a qualitative evidence synthesis
Source: BJOG. 2017 Dec 8;125(8):932–42. doi: 10.1111/1471-0528.15015 (PMC6033006; doi:10.1111/1471-0528.15015)
Supplement: Supplementary file 5 — Appendix S2. CINAHL search strategy. [file BJO-125-932-s005.pdf]

Appendix S2. CINAHL search strategy

#	Searches
S1	(MH "Maternal-Child Care+") OR (MH “Obstetric Patients+”) OR (MH “Rooming In+”) OR (MH “Obstetric Service+”) OR (MH “Childbirth+”) OR (MH "Women's Health Services/EI") OR (MH "Obstetric Care") OR TI “perinatal care” OR TI “perinatal service” OR TI “perinatal services” OR TI “peri natal care” OR TI “peri natal service” OR TI “peri natal services” OR TI “maternal care” OR TI “maternal service” OR TI “maternal services” OR TI childbirth OR TI childbirths OR AB “perinatal care” OR AB “perinatal service” OR AB “perinatal services” OR AB “peri natal care” OR AB “peri natal service” OR AB “peri natal services” OR AB “ante natal care” OR AB “ante natal service” OR AB “ante natal services” OR AB “maternal care” OR AB “maternal service” OR AB “maternal services” OR AB childbirth OR AB childbirths
S2	(MH "Health Facilities+") OR TI “Health Facility” OR TI “Health Facilities” OR TI “Medical Center” OR TI “Medical Centers” OR TI “Ambulatory Care Facility” “Ambulatory Care Facilities” OR TI “Health Center” OR TI “Health Centers” OR TI “Midwifery Service” OR TI “Midwifery Services” OR TI “Nurse-Midwifery Service” OR TI “Nurse-Midwifery Services” OR TI “Nursing Service” OR TI “Nursing Services” OR TI “Obstetric Service” OR TI “Obstetric Services” OR TI “Delivery Room” OR TI “Delivery Rooms” OR TI “Nursing Unit” OR TI “Nursing Units” OR TI “Self-Care Unit” OR TI “Self-Care Units” OR TI “Health Care Facility” OR TI “Health Care Facilities” OR TI “Hospital” OR TI “Hospitals” OR TI “Patients' Room” OR TI “Patients' Rooms” OR TI “Regional Center” OR TI “Regional Centers” OR TI “facility based” OR AB “Health Facility” OR AB “Health Facilities” OR AB “Medical Center” OR AB “Medical Centers” OR AB “Ambulatory Care Facility” OR AB “Ambulatory Care Facilities” OR AB “Health Center” OR AB “Health Centers” OR AB “Midwifery Service” OR AB “Midwifery Services” OR AB “Nurse-Midwifery Service” OR AB “Nurse-Midwifery Services” OR AB “Nursing Service” OR AB “Nursing Services” OR AB “Obstetric Service” OR AB “Obstetric Services” OR AB “Delivery Room” OR AB “Delivery Rooms” OR AB “Nursing Unit” OR AB “Nursing Units” OR AB “Self-Care Unit” OR AB “Self-Care Units” OR AB “Health Care Facility” OR AB “Health Care Facilities” OR AB “Hospital” OR AB “Hospitals” OR AB “Patients' Room” OR AB “Patients' Rooms” OR AB “Regional Center” OR AB “Regional Centers” OR AB “facility based”
S3	TI “facility based delivery” OR TI “facility based deliveries” OR TI “facility delivery” OR TI “facility deliveries” OR TI “facility based births” OR TI “facility based birth” OR “facility birth” OR TI “facility births” OR TI “clinic delivery” OR TI “clinic deliveries” OR TI “clinic births” OR TI “clinic birth” OR TI “hospital delivery” OR TI “hospital deliveries” OR TI “hospital birth” OR TI “hospital births” OR TI “hospital childbirth” OR TI “hospital childbirths” OR TI “hospital based deliveries” OR TI “hospital based delivery” OR TI “hospital based births” OR TI “institutional birth” OR TI “institutional births” OR TI “institutional childbirth” OR TI “institutional childbirths” OR TI “institutional delivery” OR TI “institutional deliveries” OR AB “facility based delivery” OR AB “facility based deliveries” OR AB “facility delivery” OR AB “facility deliveries” OR AB “facility based births” OR AB “facility based birth” OR “facility birth” OR AB “facility births” OR AB “clinic delivery” OR AB “clinic deliveries” OR AB “clinic births” OR AB “clinic birth” OR AB “hospital delivery” OR AB “hospital deliveries” OR AB “hospital birth” OR AB “hospital births” OR AB “hospital childbirth” OR AB “hospital childbirths” OR AB “hospital based deliveries” OR AB “hospital based delivery” OR AB “hospital based births” OR AB “institutional birth” OR AB “institutional births” OR AB “institutional childbirth” OR AB “institutional childbirths” OR AB “institutional delivery” OR AB “institutional deliveries”
S4	((S1 AND S2) OR S3)
S5	TI “respectful” OR AB “respectful” OR TI “confidentiality” OR TI “confidential” OR AB “confidentiality” OR AB “confidential” OR TI “informed consent” OR AB “informed consent” OR TI “human rights” OR AB “human rights” OR TI “humanization” OR TI “humanize” OR TI “humanized” OR AB “humanization” OR AB “humanize” OR AB “humanized” OR TI “humanisation” OR TI “humanise” OR TI “humanised” OR AB “humanisation” OR AB “humanise” OR AB “humanised” OR TI “dignified” TI “dignity” OR AB “dignified” AB “dignity” OR TI “medicalize” OR TI “medicalized” OR TI “medicalise” OR TI “medicalised” OR AB “medicalize” OR AB “medicalized” OR AB “medicalise” OR AB “medicalised” OR TI “empower” OR TI “empowerment” OR TI “empowered”

	OR AB “empower” OR AB “empowerment” OR AB “empowered” OR TI “quality of care” OR AB “quality of care”
S6	(MH "Patient Rights/EI/ST") OR (MH "Women's Rights/EI") OR (MH "Quality of Health Care/EI/TD") OR (MH "Patient Attitudes") OR (MH "Patient Centered Care")
S7	S4 AND (S5 OR S6)
