# Supplementary material for: Respectful care during childbirth in health facilities globally: a qualitative evidence synthesis
Source: BJOG. 2017 Dec 8;125(8):932–42. doi: 10.1111/1471-0528.15015 (PMC6033006; doi:10.1111/1471-0528.15015)
Supplement: Supplementary file 6 — Appendix S3. EMBASE search strategy. [file BJO-125-932-s006.pdf]

Appendix S3. EMBASE search strategy

	#	Searches
MATERNAL / PERINATAL HEALTH	1	'obstetric delivery':ab,ti OR 'obstetric deliveries':ab,ti OR ‘delivery’/de
	2	‘perinatal care’:ti,ab OR ‘peri natal care’:ti,ab OR ‘perinatal healthcare’:ti,ab OR ‘peri natal healthcare’:ti,ab OR ‘perinatal health care’:ti,ab OR ‘peri natal health care’:ti,ab OR 'perinatal care'/de
	3	'maternal care'/de
	4	(‘perinatal service’:ti,ab OR ‘peri natal service’:ti,ab OR ‘perinatal services’:ti,ab OR ‘peri natal services’:ti,ab OR ‘perinatal health service’:ti,ab OR ‘peri natal health service’:ti,ab OR ‘perinatal health services’:ti,ab OR ‘peri natal health services’:ti,ab OR ‘maternal care’:ti,ab OR ‘maternal health care’:ti,ab OR ‘maternal healthcare’:ti,ab OR ‘maternal service’:ti,ab OR ‘maternal health service’:ti,ab OR ‘maternal services’:ti,ab OR ‘maternal health services’:ti,ab) AND (‘birth’:ti,ab OR ‘births’:ti,ab OR ‘childbirth’:ti,ab OR ‘child birth’:ti,ab OR ‘childbirths’:ti,ab OR ‘child births’:ti,ab OR ‘delivery’:ti,ab OR ‘deliveries’:ti,ab)
	5	#1 OR #2 OR #3 OR #4
FACILITIES	6	‘birthing centers’:ti,ab OR ‘maternal-child health centers’:ti,ab OR ‘delivery rooms’:ti,ab OR ‘maternity hospitals’:ti,ab OR 'delivery room'/de
FACILITY-BASED DELIVERY	7	‘facility based delivery’:ti,ab OR ‘facility based deliveries’:ti,ab OR ‘facility delivery’:ti,ab OR ‘facility deliveries’:ti,ab OR ‘facility based births’:ti,ab OR ‘facility based birth’:ti,ab ‘facility-based childbirth’:ti,ab OR ‘facility-based child birth’:ti,ab OR ‘facility birth’:ti,ab OR ‘facility births’:ti,ab OR ‘clinic delivery’:ti,ab OR ‘clinic deliveries’:ti,ab OR ‘clinic births’:ti,ab OR ‘clinic birth’:ti,ab OR ‘hospital delivery’:ti,ab OR ‘hospital deliveries’:ti,ab OR ‘hospital birth’:ti,ab OR ‘hospital births’:ti,ab OR ‘hospital childbirth’:ti,ab OR ‘hospital childbirths’:ti,ab OR ‘hospital based deliveries’:ti,ab OR ‘hospital based delivery’:ti,ab OR ‘hospital based births’:ti,ab OR ‘institutional birth’:ti,ab OR ‘institutional births’:ti,ab OR ‘institutional childbirth’:ti,ab OR ‘institutional childbirths’:ti,ab OR ‘institutional delivery’:ti,ab OR ‘institutional deliveries’:ti,ab
	8	#5 OR #6 OR #7
Respect	9	‘Respectful’:ti,ab OR ‘dignity’:ti,ab OR ‘dignified’:ti,ab OR ‘undignified’:ti,ab OR ‘humanisation’:ti,ab OR ‘humanization’:ti,ab OR 'humanize':ab,ti OR ‘medicalize’:ti,ab OR 'medicalization':ab,ti OR ‘medicalise’:ti,ab OR 'medicalisation':ab,ti OR ‘autonomous’:ti,ab OR ‘accountable’:ti,ab OR 'accountability':ab,ti OR ‘human rights’:ti,ab OR ‘empower’:ti,ab OR 'empowered':ab,ti OR 'empowerment':ab,ti OR ‘quality of care’:ti,ab OR ‘supportive’:ti,ab OR ‘privacy’:ti,ab
	10	#8 AND (#9 OR #10)
